# Bidirectional Associations of Prosocial Behavior with Peer Acceptance and Rejection in Adolescence

**DOI:** 10.1007/s10964-022-01675-5

**Published:** 2022-09-17

**Authors:** Daniela V. Chávez, Christina Salmivalli, Claire F. Garandeau, Christian Berger, Bernadette P. Luengo Kanacri

**Affiliations:** 1 School of Psychology, Pontificia Universidad Católica de Chile, Santiago, Chile; 2 INVEST Research Flagship/Psychology, University of Turku, Turku, Finland

**Keywords:** Peer acceptance, Peer rejection, Prosocial behavior, Cross-lagged relationships, Multi-group analysis

## Abstract

As most studies on the link between peer status and prosocial behavior are cross-sectional, conducted with children, and operationalize status as the difference between acceptance and rejection, it remains unclear whether peer acceptance and rejection are consequences or prerequisites of prosocial behavior in adolescence. To fill this gap, this study examines the bidirectional associations of prosocial behavior with peer acceptance and peer rejection with data collected at 3 time points, 6 months apart, in a sample of 660 early Chilean adolescents (*M* = 12.94, SD = 0.62; 55.1% boys). Cross-lagged panel analyses showed that prosocial behavior positively predicted future peer acceptance, whereas peer acceptance had no significant effect on future prosocial behavior. The association between rejection and prosocial behavior was negative and bidirectional between Time 1 and Time 2. When a new academic year began, between Time 2 and Time 3, prosocial behavior negatively predicted rejection, whereas rejection in the previous grade level was positively associated with prosocial behavior at the beginning of the next grade. Multi-group panel analyses did not detect significant differences between boys and girls in the cross-lagged associations of prosociality with peer acceptance and peer rejection. The results suggest that acting prosocially can make adolescents better liked by their peers and highlight the possible importance of the transition to a new academic year for the prosocial behavior of previously rejected students. Implications for future research on peer relations are discussed.

## Introduction

To foster well-being at school, it is important to promote prosocial behaviors, which are voluntary actions intended to benefit others, such as sharing, helping, and comforting (Eisenberg et al., 2015). Generally, prosocial behaviors are positively associated with peer acceptance (e.g., Asher & McDonald, 2009) and negatively associated with peer rejection (e.g., Di Giunta et al., 2018). However, as most studies are cross-sectional and conducted with children, it remains uncertain whether high acceptance (and/or low rejection) is a consequence or a prerequisite of prosocial behavior, especially in adolescence. Similarly, whether low acceptance (and/or high rejection) prevents adolescents from acting prosocially or whether their lack of prosocial behavior makes their peers perceive them negatively remains to be determined. Though many studies have examined the association of prosocial behavior with social preference by using the standardized difference between acceptance (“who do you like most?”) and rejection (“who do you like least?”) scores (e.g., Berger & Rodkin, 2012; Peters et al., 2010; Sandstrom & Cillessen, 2006), some behaviors are positively associated with both acceptance and rejection (Gorman et al., 2011; Kretschmer et al., 2014). To fill these gaps, the present study uses three waves of data to examine the bidirectional associations of prosocial behavior with peer acceptance and peer rejection in a sample of early adolescents.

### Concurrent Associations Between Peer Status and Prosocial Behavior

In peer relations research, the sociometric status of each individual reflects the sentiments of peer group members towards the individual (Coie et al., 1982; Hymel et al., 2002; Dijkstra & Gest, 2015). While peer rejection (operationalized as the proportion of peers nominating them as someone they like the least) reflects the negative sentiments of peers, peer acceptance (operationalized as the proportion of peers nominating them as someone they like the most) refers to positive perceptions or feelings of affection of peers for an individual (Bukowski et al., 2000).

Research examining peer acceptance and rejection separately has consistently shown that children who are well-liked by their peers are more likely to behave prosocially (Closson & Hymel, 2016; Dijkstra & Gest, 2015; Findley-Van Nostrand & Ojanen, 2018; Wentzel & McNamara, 1999), and those who are rejected are less likely to behave prosocially (Caputi et al., 2012). Likewise, the composite score of “social preference” (i.e., subtracting liked least from liked most) is positively associated with prosocial behavior (Sandstrom & Cillessen, 2006; Wolters et al., 2014). However, the social preference measure (i.e., the standardized difference between acceptance and rejection) is limited in that scores close to zero might indicate that a student is both highly liked and highly rejected (i.e., controversial) or neither liked nor rejected (i.e., neglected). Research has shown that children with a controversial status and those with a neglected status have very different behavioral profiles (Newcomb et al., 1993). Thus, using a measure that does not distinguish the two status indices (acceptance and rejection) could hide possible effects of each type of status on prosocial behavior, failing to provide an accurate picture of the status-prosocial behavior links.

In sum, from concurrent evidence, it is not clear whether being liked and accepted or rejected and excluded by peers influence adolescents’ prosocial behaviors or whether being prosocial predicts future evaluations by peers.

### Prospective Effects of Peer Status on Prosocial Behavior

Research on the prospective effects of peer status on prosocial behavior among children and adolescents is scarce and has yielded inconsistent findings. In a sample of Chinese adolescents, a higher level of peer acceptance was found to predict higher levels of prosocial behavior after controlling for academic ability (Lu et al., 2018). The authors emphasized that prosocial behavior tends to be more normative in collectivistic cultural contexts, such as China. Thus, peer status could be differently linked to prosocial behavior in other, more individualistic, contexts. Indeed, other longitudinal studies did not find support for the positive association. A study conducted with Australian children aged 5 to 7 showed that social preference was not significantly associated with their prosocial behavior two years later (Kuhnert et al., 2017). In an Italian sample of the same age, a longitudinal study found no significant effect of either acceptance or rejection on prosocial behavior one year later (Caputi et al., 2012).

On the one hand, being liked should promote prosocial behavior because well-accepted adolescents are more likely to experience positive social interactions with their peers, and receiving positive peer treatment should increase their motivation to respond in kind. Indeed, experimental research with adults has found evidence for reciprocity in prosocial behavior, with participants choosing to be more generous to those who had previously helped them (e.g., see Krockow et al., 2016). Moreover, well-accepted youth are more likely to have friends (Gifford-Smith & Brownell, 2003; Son & Padilla-Walker, 2020), providing them with more opportunities for being helpful, cooperative, and sharing. On the other hand, because those with high levels of acceptance are likely to already enjoy frequent positive social interactions and have many friendships fulfilling their belonging needs (Baumeister & Leary, 1995), they may not have an incentive to increase their prosocial behavior.

Similarly, highly rejected adolescents are more likely to experience social exclusion from peers (Buhs & Ladd, 2001) and therefore are deprived of opportunities to act in prosocial ways (Parker et al., 2006; Twenge et al., 2007). Moreover, experimental research with undergraduate students suggests that experiencing social exclusion in a laboratory setting (e.g., I do not want to work with this person) evokes stronger negative responses such as anger, hurt, sadness, and lower happiness than experiencing acceptance (Buckley et al., 2004). In turn, these emotions could inhibit prosocial actions. A meta-analysis of experimental studies has shown that peer rejection makes people feel bad and leads to a tendency to behave aggressively towards the rejector in order to restore control (Gerber & Wheeler, 2009). Therefore, being rejected by peers might hinder prosocial behavior towards them. However, there is also evidence among undergraduate students suggesting that social exclusion might motivate individuals to seek new friends and thus increase their prosocial behavior (Maner et al., 2007). Socially excluded individuals aiming to restore connections and belonging may resort to prosocial behaviors to achieve this goal (DeWall & Richman, 2011).

### Prospective Effects of Prosocial Behavior on Peer Status

Regarding the effects of prosocial behavior on future acceptance and rejection, findings are mixed. An experimental study prompting 9- to 11-year-olds to perform prosocial acts found that it increased their level of peer acceptance (Layous et al., 2012). A longitudinal study found positive links between prosocial behavior and future acceptance in a cross-lagged panel model in a sample of Chinese adolescents (Lu et al., 2018). Although these two studies conducted with children and adolescents indicate that prosocial behavior can increase their acceptance among peers, several experiments conducted with young adults—demonstrate that it may not always be the case.

Indeed, recent findings have shown that prosocial behavior could even decrease peer acceptance. In an experiment with university students, being outstandingly prosocial was found to decrease acceptance (e.g., likeability) and provoke rejection (Boileau et al., 2021). Likewise, using four different data sets, it has been demonstrated that individuals who were particularly generous in a public goods game became excluded by the group (Parks & Stone, 2010). These negative effects of prosociality on status are explained by the fact that exceptionally generous individuals set a high standard for behavior, making peers look bad in comparison and thereby eliciting rejection (Boileau et al., 2021; Parks & Stone, 2010). Therefore, it is not yet clear whether behaving prosocially leads to more acceptance or rejection.

### Gender and Developmental Considerations

In contrast with the development of antisocial behavior (e.g., aggression) that tends to peak in adolescence, partly because it relates to competition for high popularity among peers (Casper et al., 2020; Prinstein & Cillessen, 2003), developmental studies on prosocial behavior have shown the opposite trend. These studies have suggested a normative decline in mean levels of (self-reported) prosocial behavior from early to middle adolescence for boys and girls (Carlo et al., 2007); this decline persists until age 17 before prosocial behavior increase again until age 21 (Luengo Kanacri et al., 2013). For this reason, early adolescence is a particularly important developmental transitional phase for examining longitudinally mechanisms that might promote prosociality in the long term.

The present study also explores the moderating role of gender as literature has reported important gender differences in developmental processes in adolescence, with girls being more accepted (Wentzel & McNamara, 1999) and likely to engage in prosocial interactions, endorse connection-oriented goals, and seek support (Rose & Rudolph, 2006). In contrast, boys usually receive more nominations as rejected compared to girls (Coie et al., 1982), tend to emphasize the importance of dominance goals and self-interest, are more prone to engage in competitive play, and have more exposure to aggressive behavior and victimization by peers (Rose & Rudolph, 2006). Therefore, longitudinal research on the links between peer status and prosocial behavior is needed to clarify the dynamics of these associations from a developmental perspective, while also exploring gender differences over time.

## Current Study

To clarify whether adolescents’ prosocial behavior predicts their peer status, whether their peer status has an effect on their prosocial behavior, and whether these prospective associations are positive or negative, this study tests the bidirectional associations between two measures of peer status -acceptance and rejection—and prosocial behavior across three waves. Most research on the topic has not disentangled the direction of effects between peer status and prosocial behavior as longitudinal studies on these associations are scarce, and few were conducted with adolescents. The present study also explores the moderating role of gender in these associations as the literature has reported important gender differences in this developmental phase. As prior findings have been mixed, no directional hypothesis is formulated for these prospective, bidirectional links.

## Method

### Participants

The present study uses longitudinal data collected as part of a larger study assessing an educational intervention aimed at promoting prosocial behaviors and civic engagement among adolescents in Santiago, Chile: the ProCiviCo Intervention Project (for details, see Luengo Kanacri & Jiménez-Moya, 2017; Luengo Kanacri et al., 2020). The data used in this study includes the first three waves of the ProCiviCo project, namely, the pre-intervention data collected in May 2017 (nT1 = 660) when students attended 7^th^ grade, and the next two follow-up assessments, collected in October 2017 and in May 2018 (T2) when students were in Grade 7 and 8, respectively (*M*_age_ = 12.29, SD = 0.62; 55.1% males). Participating schools were selected according to socioeconomic heterogeneity criteria and then were randomly assigned to the intervention (four schools; *N* = 324) and control (four schools; *N* = 336) conditions. The participation rate in this study was over 90% across classrooms (ranging from 99.4% at T2 to 94.5% at T3). In total, 16 classrooms participated in the study where 26% of the students belonged to the low-middle class, 21% middle class, 9% to the low class, and 0.5% belonged to the middle-high class. For 43.5%, the information on SES was not available.

### Procedure

Letters were sent home with children describing the purpose of the study, and parental informed consent was obtained at each assessment point while children’s assent was ensured. Questionnaires and peer reports for students were administered in each classroom by three to four members of the research team during school hours. The response choices of the questionnaires were explained to students during data collection. All instruments and procedures were approved by the ethics committee at the Catholic University of Chile and by the Chilean National Funding of Science and Technology (FONDECYT).

### Measures

#### Prosocial behavior

Peer ratings were used to measure prosocial behavior. At each wave, the participating students were asked to rate the frequency of four types of prosocial behavior (“He/she tries to comfort other classmates when they are sad”; “shares with others things he/she likes”, “He/she tries to understand the point of view of others”; “He/she helps others who are in need or have problems”) displayed by each of their classmates on a five-point scale ranging from 1 (never) to 5 (almost always). A score of prosocial behavior was computed for each individual by averaging the ratings they received from all nominators. Cronbach’s alpha showed high internal consistency at each time point (T1 *α* = 0.96; T2 *α* = 0.95; T3 *α* = 0.95).

#### Peer acceptance

One peer nomination question was used to measure peer acceptance (Bellmore & Cillessen, 2003; Cillessen & Marks, 2017). At each wave, students were presented with a roster of their classmates and asked to nominate up to three classmates who best fit the description: “With whom would you like to hang out at school during recess?” For each student, a proportion score of peer acceptance was calculated by dividing the total number of nominations received by the total number of students within each classroom (i.e., the total number of possible nominations). Cross-gender nominations were allowed.

#### Peer rejection

One peer nomination question was used to measure peer rejection. Students nominated up to three classmates at each wave by answering: “With whom would you not like to hang out at school during recess?” Cross-gender nominations were allowed. The scores were computed using the same procedure that was used for the peer acceptance scores.

### Control Variables

As the participants were selected from a population characterized by high levels of inequality and segregation (Luengo Kanacri & Jiménez-Moya, 2017), which could potentially undermine the development of cooperation, this study controlled for socioeconomic status (SES) in all the models. In the first set of models where no constraints were applied, SES, gender, and intervention status were added as control variables. Participants’ SES was reported by their parents at T1. Parents answered the question “to which socioeconomic class does your family belong?” on a five-point scale, ranging from low to high class. Given that no one reported belonging to the high socioeconomic status group and only three cases reported being in the middle-high category, a dummy code was created for SES, grouping the low SES with low-middle (coded as 1), and the middle SES with middle-high (coded as 0). The gender of the students was coded as 0 = girls, 1 = boys. Since the data was collected as part of the evaluation of an intervention designed to promote prosocial behavior, group condition (intervention vs control) was also controlled for in all the models.

### Analytical Strategy

To examine bidirectional associations between peer status—acceptance and rejection—and prosocial behavior, a three-wave cross-lagged panel model (CLPM) was estimated using the Lavaan package in R (Rosseel, 2012). Missing data across the three waves (0.6% at T2 and 5.5% at T3) were handled using Full information maximum-likelihood (FIML), as it offers less biased estimates even when the pattern of missingness cannot be ignored (Baraldi & Enders, 2010).

In the first step, two separate models were estimated for peer acceptance and peer rejection. Gender, SES, and group condition (intervention vs. control) were controlled for by regressing prosocial behavior and peer status variables at each time point on them. Additionally, second-order autoregressive paths were included in the model (Little, 2013), representing delayed effects across the associations of prosocial behavior with peer acceptance and rejection from T1 to T3 (Newsom, 2015).

Second, a multi-group path analysis for gender was conducted to estimate potential differences between boys and girls in the cross-lagged associations. The multi-group analysis started with the estimation of a freely estimated multi-group model (Model 0) where all parameters were estimated without constraints, controlling for experimental condition and SES. Then, two subsequent models were estimated where: a) the autoregressive parameters were constrained to be equal (Model 1), and b) both autoregressive and cross-lagged parameters were constrained to be equal (Model 2).

Third, in order to compare models, the Bayesian Information Criterion (BIC) was used, which is known to be less sensitive to sample size (Meade et al., 2008; West et al., 2012). A lower BIC indicates a better model in terms of model fit and model complexity (i.e., number of parameters). First, Model 0 was compared with Model 1, and the best fitting model (smaller value meaning no differences) was compared with Model 2, in which gender differences in the cross-lagged associations of prosocial behavior with peer acceptance and rejection were tested.

Goodness of fit for all the models was evaluated using the indices that are also less sensitive to sample size (Kline, 2011). The comparative fit index (CFI), the Tucker-Lewis index (TLI), and the root mean square error of approximation (RMSEA). CFI and TLI range from 0 to 1; values greater than 0.90 and 0.95 are indicative of acceptable and good model fit. The RMSEA ranges from 0 to 1 (<0.05 indicates good fit; <0.08 indicates acceptable fit, with associated 95% confidence intervals (CIs) (see Brown, 2015).

Because the data used in this study came from the evaluation of an intervention designed to promote prosocial behavior among early adolescents, a sensitivity analysis was conducted. More specifically, an additional multigroup analysis was estimated to test whether the cross-lagged paths hypothesized in this study were significantly different in the intervention group and in the control group. Finally, the cross-lagged models for acceptance and rejection using only the participants from the control group were also tested.

## Results

### Descriptive Statistics and Correlations

Means and standard deviations for the study variables are presented in Table 1 separately for boys and girls. Significant gender differences in prosocial behavior were found at each time point (*p*s < 0.001), with girls scoring higher than boys. No gender differences were found for acceptance or rejection. The bivariate correlations for the main variables are presented in Table 2. At each time point, prosocial behavior was positively associated with peer acceptance and negatively associated with peer rejection for both boys and girls. The association between the two measures of status, peer acceptance and rejection, was very low. It was negative at T1 (*r* = −0.15 for boys and *r* = −0.13 for girls) and at T2 for girls (*r* = −0.14). It was non-significant at T2 for boys (*r* = −0.06) and at T3 for both boys (*r* = 0.01) and girls (0.00). These correlations demonstrate that the overlap between acceptance and rejection is indeed limited, supporting the decision to examine the two variables separately.

**Table 1:** Means and standard deviations for boys and girls

Variable	Full sample	Boys (*N* = 363)	Girls (*N* = 297)	*t*-test
Time 1 (*N* = 660)				
Prosocial behavior	3.02 (0.55)	2.86 (0.52)	3.21 (0.53)	−8.11***
Peer acceptance	0.06 (0.05)	0.06 (0.05)	0.07 (0.05)	−1.33
Peer rejection	0.06 (0.08)	0.06 (0.08)	0.07 (0.08)	−0.78
Time 2 (*N* = 656)				
Prosocial behavior	3.07 (0.54)	2.93 (0.51)	3.24 (0.54)	−7.02***
Peer acceptance	0.06 (0.06)	0.06 (0.06)	0.07 (0.05)	−0.94
Peer rejection	0.06 (0.08)	0.07 (0.08)	0.07 (0.08)	−0.22
Time 3 (*N* = 624)				
Prosocial Behavior	3.15 (0.50)	3.01 (0.46)	3.37 (0.47)	−8.67***
Peer acceptance	0.06 (0.06)	0.06 (0.06)	0.06 (0.06)	0.25
Peer rejection	0.06 (0.08)	0.07 (0.08)	0.06 (0.08)	1.52

*M* mean, *SD* standard deviation

***p < 0.001

**Table 2:** Bivariate associations across time and for boys and girls

Variables	1	2	3	4	5	6	7	8	9
1. Prosociality T1		0.81^a^	0.84^a^	0.24^a^	0.26^a^	0.29^a^	−0.55^a^	−0.50^a^	−0.34^a^
2. Prosociality T2	0.84^a^		0.85^a^	0.21^a^	0.28^a^	0.28^a^	−0.48^a^	−0.56^a^	−0.36^a^
3. Prosociality T3	0.78^a^	0.89^a^		0.18^a^	0.28^a^	0.35^a^	−0.40^a^	−0.46^a^	−0.54^a^
4. Acceptance T1	0.32^a^	0.25^a^	0.27^a^		0.55^a^	0.40^a^	−0.13^b^	−0.09	−0.06
5. Acceptance T2	0.24^a^	0.23^a^	0.26^a^	0.50^a^		0.39^a^	−0.22^a^	−0.14^a^	−0.16^a^
6. Acceptance T3	0.19^a^	0.20^a^	0.27^a^	0.48^a^	0.49^a^		−0.25^a^	−0.16^a^	−0.00
7. Rejection T1	−0.47^a^	−0.45^a^	−0.41^a^	−0.15^a^	−0.15^a^	−0.15^a^		0.66^a^	0.42^a^
8. Rejection T2	−0.34^a^	−0.45^a^	−0.39^a^	−0.13^b^	−0.06	−0.05	0.68^a^		0.52^a^
9. Rejection T3	−0.31^a^	−0.41^a^	−0.47^a^	−0.12^b^	−0.08	0.01	0.54^a^	0.67^a^	

Correlations for girls are presented at the top triangle, while correlations for boys are presented at the bottom triangle. Total sample T1 = 660; T2 = 656; T3 = 624

^a^Correlations are significant at 0.01

^b^Correlations are significant at 0.05

### Bi-Directional Associations Between Peer Status and Prosocial Behavior

#### Peer acceptance

**Fig. 1:**
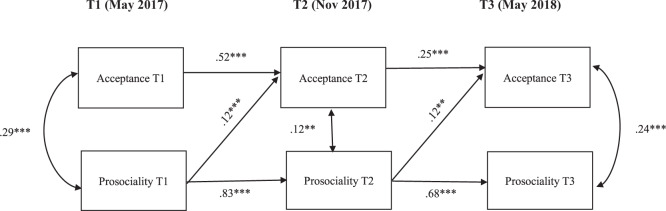
Cross-lagged panel model (CLPM) of peer-reported acceptance and prosocial behavior, controlled by gender, SES, and group condition (intervention vs. control group) at all time points. Only pathways with significant standardized estimates are shown in the figure. *p < 0.05, **p < 0.01, ***p < 0.001

The cross-lagged panel model testing the associations between peer acceptance and prosocial behavior across three time points (Fig. 1) had a good fit, *X*^2^(2) = 0.750, *p* = 0.687, CFI = 1.000, TLI = 1.010, RMSEA = 0.000 (90% confidence interval = 0.000–0.057). To improve the model fit, second-order autoregressive paths were included in the model (Little, 2013). Thus, T3 prosocial behavior was regressed on T1 prosocial behavior, and T3 peer acceptance was regressed on T1 peer acceptance.

Autoregressive paths for both acceptance and prosocial behavior were positive and significant across time (*p*s < 0.001). Prosocial behavior at T1 was positively associated with peer acceptance at T2 (*β* = 0.12, *p* = 0.001), and prosocial behavior at T2 was positively associated with peer acceptance at T3 (*β* = 0.12, *p* = 0.002), suggesting that engaging in more prosocial behavior led adolescents to be better-liked by their peers. However, there was no indication that this effect would be bidirectional. Peer acceptance at T1 did not predict prosocial behavior at T2, and peer acceptance at T2 did not predict prosocial behavior at T3. For the significant paths from prosocial behavior to peer acceptance over time, the effect of control variables, such as SES and gender, showed no significant effect. Meanwhile, being in the intervention group showed a positive and significant effect on peer acceptance at T2 (*β* = 0.11, *p* = 0.001), and at T3 (*β* = 0.12, *p* = 0.002).

#### Peer rejection

**Fig. 2:**
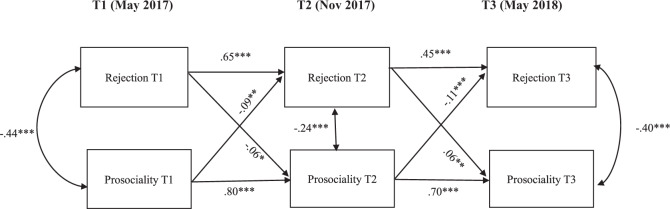
Cross-lagged panel model (CLPM) of peer-reported rejection and prosocial behavior, controlled by gender, SES, and group condition (intervention vs. control group) at all time points. Only pathways with significant standardized estimates are shown in the figure. *p < 0.05, **p < 0.01, ***p < 0.001

The cross-lagged panel model testing the associations between peer rejection and prosocial behavior is presented in Fig. 2. This model had a good fit, *X*^2^(2) = 9.35, *p* = 0.096, CFI = 0.998, TLI = 0.987, RMSEA = 0.037 (90% confidence interval = 0.000–0.074). Similar to the previous model, second-order autoregressive paths were included to improve the model fit (Little, 2013). Specifically, the effects of T1 prosocial behavior on T3 prosocial behavior, and of T1 peer rejection on T3 peer rejection were added to the model.

Results show that prosocial behavior at T1 was negatively associated with peer rejection at T2 (*β* = −0.09, *p* = 0.008), and prosocial behavior at T2 was negatively associated with peer rejection at T3 (*β* = −0.11, *p* = 0.006). Moreover, between T1 and T2, this effect was bidirectional, with T1 peer rejection negatively predicting T2 prosocial behavior (*β* = −0.06, *p* = 0.025). However, peer rejection at T2 positively predicted prosocial behavior at T3 (*β* = 0.06, *p* = 0.022). The autoregressive paths from Time 1 to Time 3 were all significant for both peer rejection and for prosocial behavior (*p* < 0.001).

For the significant paths from prosocial behavior to peer rejection over time, the effect of control variables, such as SES, gender, and group intervention showed no significant effect at any time point. Meanwhile, significant effects of gender and SES on prosocial behavior at T3 were found. More specifically, there was a negative effect for boys (*β* = −0.94, *p* < 0.001), and a positive effect for the low-middle class (*β* = 0.07, *p* = 0.014) at T3. For the effect of group condition, results only showed a significant and negative effect of the intervention on prosocial behavior at T2 (*β* = −0.07, *p* = 0.004).

### Gender Differences

#### Peer acceptance

Multi-group analyses were conducted to examine whether the bidirectional effects between peer acceptance and prosocial behavior (cross-lagged paths) differed for boys and for girls (see Table 3). In these set of models, the same second-order autoregressive paths were included to improve model fit (Little, 2013) while controlling for SES and experimental condition. The freely estimated model (Model 0) had a good fit, *X*^2^(4) = 1.82, *p* = 0.770, CFI = 1.000, TLI = 1.016, RMSEA = 0.000 (90% confidence interval = 0.000–0.059), and it was compared to a model where only autoregressive paths were set equal for girls and boys (Model 1) which had also a good fit, *X*^2^(8) = 14.67, *p* = 0.066, CFI = 0.995, TLI = 0.967, RMSEA = 0.062 (90% confidence interval = 0.000–0.111). A lower BIC value in Model 1 (BIC = −3064.0), where all autoregressive paths were left constrained, indicated that this model had a better fit in comparison with Model 0 (BIC = −3056.7), where all paths were estimated freely. Thus, for the next comparison, autoregressive paths were left constrained across gender since no gender differences were supported in Model 1.

**Table 3:** Test of model fit and chi-square different test by applying different equality constraints for peer acceptance and prosocial behavior for boys and girls

Constraints	*X*^2^ (df)	CFI	TLI	RMSEA	BIC	Model comparison
Model 0: freely estimated	1.82 (4)	1.000	1.016	0.000	−3056.7	
Model 1: autoregressive	14.67 (8)	0.995	0.967	0.062	−3064.0	1 vs. 0
Model 2: auto + cross-lagged	19.67 (12)	0.995	0.978	0.051	−3085.7	2 vs. 1

*X*^*2*^ chi-square, *df* degrees of freedom, *CFI* comparative fit index, *TLI* Tucker–Lewis index, *RMSEA* root mean square error of approximation, *BIC* Bayesian information criterion

Finally, to assess gender differences in the cross-lagged associations between peer acceptance and prosocial behavior, Model 2 was estimated constraining both autoregressive and cross-lagged paths between boys and girls and leaving the selected Model 1 as the model for comparison. This second model also presented a good fit, *X*^2^(12) = 19.673, *p* = 0.074, CFI = 0.995, TLI = 0.978, RMSEA = 0.051 (90% confidence interval = 0.000–0.090). The lower BIC value in Model 2 (BIC = −3085.7) in comparison with Model 1 (BIC = −3064.0) indicated that Model 2 was the model selected, and no gender differences between boys and girls were found in the cross-lagged associations between peer acceptance and prosocial behavior.

#### Peer rejection

Results of the multigroup analyses testing for gender differences in the bidirectional associations between peer rejection and prosocial behavior are shown in Table 4. To improve the model fit, the second-order autoregressive paths were included in the model (Little, 2013), while controlling for SES and experimental condition. The first multi-group and freely estimated model (Model 0) had a good fit, *X*^2^(4) = 3.68, *p* = 0.451, CFI = 1.000, TLI = 1.002, RMSEA = 0.000 (90% confidence interval = 0.000–0.081). When constraining the autoregressive paths (Model 1), this model also had an adequate fit, *X*^2^(8) = 16.15, *p* = 0.040, CFI = 0.994, TLI = 0.960, RMSEA = 0.076 (90% confidence interval = 0.015–0.129). When comparing Model 0 and Model 1, the smaller BIC value in Model 1 (BIC = −2033.7) in comparison with Model 0 (BIC = −2032.0), suggested that the autoregressive paths are all equal across gender, and Model 1 is the model selected.

**Table 4:** Test of model fit and chi-square different test by applying different equality constraints for peer rejection and prosocial behavior for boys and girls

Constraints	*X*^2^ (df)	CFI	TLI	RMSEA	BIC	Model comparison
Model 0: freely estimated	3.68 (4)	1.000	1.002	0.000	−2032.0	
Model 1: autoregressive	16.15 (8)	0.994	0.960	0.076	−2033.7	1 vs. 0
Model 2: auto + cross-lagged	24.95 (12)	0.992	0.963	0.072	2050.3	2 vs. 1

*X*^*2*^ chi-square, *df* degrees of freedom, *CFI* comparative fit index, *TLI* Tucker–Lewis index, *RMSEA* root mean square error of approximation, *BIC* Bayesian information criterion

Finally, the model where both autoregressive and cross-lagged paths were left constrained (Model 2) presented a good fit, *X*^2^(12) = 24.95, *p* = 0.015, CFI = 0.992, TLI = 0.963, RMSEA = 0.072 (90% confidence interval = 0.031–0.112). The lower BIC value in Model 2 (BIC = −2050.3) in comparison with Model 1 (BIC = −2033.7) suggested that Model 2 was the best model, and no gender differences were found in the cross-lagged associations between peer rejection and prosocial behavior.

### Sensitivity Analyses

Acknowledging that the main goal of this study was to test the longitudinal associations between prosocial behavior and peer status (acceptance and rejection), it is important to note that the data used in the analyses comes from the evaluation of an intervention designed to promote prosocial behavior among students. For this reason, the intervention condition was a control variable in all models, and furthermore, a multi-group analysis was conducted to examine whether the cross-lagged associations between the main variables of this study differed between the intervention group and the control group.

This was tested with the same procedure used for testing gender differences in the previous models, but in this case, using the group condition (intervention vs. control) as the grouping variable, while controlling for gender and SES. In the peer acceptance model, the three models estimated had a good fit, Model 0, *X*^2^(4) = 3.61, *p* = 0.462, CFI = 1.000, TLI = 1.002, RMSEA = 0.000 (90% confidence interval = 0.000–0.0078); Model 1, *X*^2^(8) = 5.98, *p* = 0.649, CFI = 1.000, TLI = 1.008, RMSEA = 0.000 (90% confidence interval = 0.000–0.059), and Model 2, *X*^2^(12) = 11.11, *p* = 0.520, CFI = 1.000, TLI = 1.002, RMSEA = 0.000 (90% confidence interval = 0.000–0.057). When comparing the first two models, the lower BIC value in Model 1 (BIC = −3649.8) compared to Model 0 (BIC = −3627.9) indicated that the autoregressive paths were equal across intervention and control groups. Likewise, the lower BIC value in Model 2 (BIC = −3670.5) in comparison to Model 1 (BIC = −3649.8) suggested that the cross-lagged associations of prosociality with peer acceptance were equal across intervention and control groups. Therefore, no evidence was found for group differences (intervention/control) in the effect of prosocial behavior on future acceptance or the effect of peer acceptance on future prosocial behavior.

For the peer rejection model, the three estimated models presented a good fit, Model 0, *X*^2^(4) = 4.51, *p* = 0.342, CFI = 1.000, TLI = 0.997, RMSEA = 0.020 (90% confidence interval = 0.000–0.090); Model 1, *X*^2^(8) = 5.84, *p* = 0.665, CFI = 1.000, TLI = 1.010, RMSEA = 0.000 (90% confidence interval = 0.000–0.069), and Model 2, *X*^2^(12) = 14.65, *p* = 0.261, CFI = 0.998, TLI = 0.993, RMSEA = 0.032 (90% confidence interval = 0.000–0.080). When comparing the first two models, the lower BIC value in Model 1 (BIC = −2501.1) compared to Model 0 (BIC = −2480.5), indicated that the autoregressive paths were equal across intervention and control groups. Likewise, the lower BIC value in Model 2 (BIC = −2514.7) in comparison to Model 1 (BIC = −2501.1) suggested that the cross-lagged associations of prosociality with peer rejection were equal across intervention and control groups.

In sum, none of the cross-lagged paths presented in this study regarding the bidirectional associations of prosocial behavior with peer acceptance (Fig. 1), and with peer rejection (Fig. 2) was affected by the intervention.

Finally, the cross-lagged panel model was estimated with the control group participants only, controlling for gender and SES. The models for acceptance and rejection had a good fit, *X*^2^(2) = 2.74, *p* = 0.254, CFI = 0.999, TLI = 0.990, RMSEA = 0.033 (90% confidence interval = 0.000–0.120), and *X*^2^(2) = 0.79, *p* = 0.673, CFI = 1.000, TLI = 1.015, RMSEA = 0.000 (90% confidence interval = 0.000–0.090), respectively. The models showed that, although the directions of the cross-lagged associations were similar to the ones found in the whole sample, minor differences were found in the strength of these associations. For instance, the estimates (standardized betas) were slightly stronger in the following paths: from prosocial behavior to peer acceptance T1–T2, *β* = 0.24, *p* < 0.001; and T2–T3, *β* = 0.15, *p* = 0.028, from prosocial behavior to peer rejection T1–T2, *β* = −0.12, *p* < 0.05; and T2–T3, *β* = −0.21, *p* < 0.001, and from peer rejection to prosocial behavior T1–T2, *β* = −0.12, *p* = 0.005. Meanwhile, the positive path from T2 rejection to T3 prosocial behavior was not significant (*β* = 0.06, *p* > 0.05), which may be related to the smaller size of the sample (*N* = 324).

## Discussion

Developmental psychologists have long acknowledged that students who behave prosocially tend to be well-liked and not to be disliked by their peers (e.g., Di Giunta et al., 2018). However, the lack of longitudinal studies, especially in adolescence, on the bidirectional associations of prosocial behavior with sociometric peer status—examining both acceptance and rejection separately—made it difficult to understand how prosocial behavior and peer status might influence each other over time. Moreover, some research has questioned the positive association between acceptance and prosociality, suggesting that outstanding prosocial individuals may elicit rejection from others by making them look bad in comparison (e.g., Parks & Stone, 2010). Other recent research has suggested that when social exclusion leads to feeling disliked, individuals might increase their prosocial behavior in response (Debono et al., 2020). To clarify how behaving prosocially relates to being liked and being disliked in adolescence, this study tested the longitudinal, bidirectional associations of prosocial behavior with peer acceptance and peer rejection using three waves of data.

The results of this study support the concurrent positive association between peer acceptance and prosocial behavior, and the concurrent negative association between peer rejection and prosocial behavior, well-established in the literature using cross-sectional data (e.g., Closson & Hymel, 2016). The findings that acceptance and rejection had a low correlation with one another and were differentially associated with prosocial behavior with regard to the strength of the association, supported the idea that these two types of status should not be understood as opposite ends of a continuum that range from acceptance to rejection (e.g., social preference), but rather as distinct experiences.

With regard to the longitudinal associations between peer acceptance and prosocial behavior, the cross-lagged panel analysis showed that prosocial behavior positively predicted peer acceptance over time, but peer acceptance did not predict future prosocial behavior. Contrary to some findings suggesting that, in some cases, prosocial behavior can elicit dislike from others (e.g., Boileau et al., 2021), the results of this study show that engaging in friendly, kind behaviors such as sharing, helping, or comforting others, helps adolescents gain more acceptance from their peers. However, no evidence was found that being well-liked led to increases in prosocial behavior. Although being already well-accepted should logically provide adolescents with more opportunities to behave prosocially, well-liked students might not increase their prosocial behavior over time, either because they are satisfied with their status and do not feel the need to gain additional acceptance via prosocial behavior, or because they are already highly prosocial and have no room to increase (i.e., ceiling effect). As suggested by Dijkstra et al. (2015), the behavior of accepted adolescents is strongly motivated by hedonic goals, meaning that they seek direct gratification by feeling good, being spontaneous, and being “fun-seeking”, which might explain their high levels of acceptance, but it does not explain prosocial behavior as a consequence of that acceptance.

With regard to the longitudinal links between prosocial behavior and peer rejection, evidence of a bidirectional association was found. The less adolescents engaged in prosocial behavior, the more rejected they became. The negative prospective link between prosocial behavior and peer rejection was stronger from T2 to T3, when students transitioned to a new academic year. Being rejected predicted decreases in prosocial behavior from T1 to T2 but increases in prosocial behavior from T2 to T3, meaning that adolescents who were disliked at the end of one academic year (T2), displayed more prosocial behavior at the beginning of the new academic year (T3). Those who are rejected by their peers might see the beginning of a new academic year as an opportunity to reconnect with the rest of the group and foster new friendships. Therefore, they might decide to display more prosocial behavior in an attempt to change their peers’ perceptions of them and decrease their aversion (Cuadrado et al., 2016). This explanation is consistent with prior studies suggesting that earlier rejection can increase prosociality (e.g., DeWall & Richman, 2011). As low-status students might be more willing to consider a positive behavioral change at the beginning of the academic year, this might be the optimal time for adult efforts to reinforce cooperative interactions among peers.

The major strengths of this study are its 3-wave longitudinal design and its separate examination of peer acceptance and rejection. The weak correlations between these two variables in the present study for both girls and boys support the idea that analyzing the two constructs is more appropriate than examining their composite score based on the standardized difference (Marks et al., 2021). Another strength is the use of peer reports to assess not only peer status but also behavior. Unlike self-reports, peer-reported measures rely on multiple informants and are not influenced by socially desirable responding, thus capturing the *strength of the reputation* of being prosocial.

This study also has some limitations that should be considered. First, the number of nominations for the peer status measures was limited to only three classmates, which could artificially limit the selection of other peers who might also fit the description for acceptance and rejection. Therefore, some participants may have obtained higher acceptance or rejection scores if unlimited nominations had been allowed.

Second, the present study only considered between-person associations, meaning that all the cross-lagged associations estimated in the analyses accounted for differences between students but did not show whether and how changes over time for each individual might affect reciprocal associations between status and prosocial behavior. Analyses of within-person associations might have yielded slightly different results. For instance, they might have revealed that becoming more accepted than one’s average level of acceptance leads adolescents to behave more prosocially than usual. However, within-person associations are more difficult to interpret when using only peer-nominated variables, as scores obtained from peer nominations tend to reflect between-person differences. Future studies might consider the use of self-report measures when examining these longitudinal associations while separating between- from within-person variance. Alternatively, it might be that only adolescents with some specific levels of acceptance (e.g., low or medium) increase in prosociality, which could be why the analyses did not detect a positive effect of acceptance on later prosociality. Finally, the between-school variance was not controlled for in the analyses, even though the dynamic associations between status and prosociality might, to some extent, vary depending on the school context (for example, SES level, as well as the degree of SES inequality, might differ across schools). Future studies might consider the nested structure of schools and classrooms to assess these potential differences in the associations between status and prosocial behavior.

When interpreting the findings of this study, it should be kept in mind that the effect sizes were small. Moreover, this study was conducted in a sample of early Chilean adolescents from a highly segregated and unequal context in terms of socioeconomic background and educational system, which is considered a challenging context for the development of cooperation and social cohesion (Luengo Kanacri & Jiménez-Moya, 2017). Although parent-reported SES was controlled for in all models, it is still possible that other social characteristics such as ethnic background, neighborhood, or parental education, also affected the way in which acceptance and rejection predict prosocial behavior.

It should be noted that data for the present study was drawn from a larger project assessing the efficacy of an educational intervention to promote social cohesion and prosocial behavior among peers. It is therefore possible that the intervention made adolescents aware of the role of prosocial behavior in peer relations and adjustment (Ripoll-Núñez et al., 2020). Acknowledging prosocial behavior as a driver for social acceptance—and highlighting the relevance of acceptance as an indicator of social status—should be a key component of school interventions. Moreover, providing rejected adolescents with opportunities to share with others (for instance, in extracurricular activities) might foster their integration by means of displaying prosocial behaviors, thus lighting up a positive reinforcing cycle of social acceptance and prosociality.

## Conclusion

As most studies associating prosocial behavior with peer acceptance and rejection were cross-sectional, conducted with children or adults, and operationalized status as the difference between acceptance and rejection, this study used a longitudinal design to investigate whether both peer acceptance and peer rejection are consequences or predictors of prosocial behavior in adolescence. Behaving in a prosocial manner (such as being kind, helping, caring, and sharing) was found to increase peer acceptance and decrease peer rejection in early adolescents. This study confirms that (prosocial) behavior can predict peer status, both acceptance and rejection; but the effects of status on prosocial behavior are less clear. Acceptance did not significantly predict prosocial behavior at any time point, and rejection negatively predicted prosocial behavior during the same school year, but positively predicted it across the transition to a new school year. As the beginning of a new academic year might represent a “fresh start” for rejected adolescents and an opportunity to behave more prosocially, future studies could assess some potential mediators of this association. For instance, the degree to which rejected adolescents perceive themselves as being rejected could determine their future prosocial actions. Thus, interventions to promote well-being and adaptive development in adolescence should focus on the beneficial role of prosocial behaviors for peer relationships, especially at the beginning of each academic year, to help rejected peers to better connect with their classmates and be more socially included.
